# Seasonality in mood disorders: Probing association of accelerometer-derived physical activity with daylength and solar insolation

**DOI:** 10.1371/journal.pmen.0000124

**Published:** 2024-09-25

**Authors:** Oleg Kovtun, Sandra J. Rosenthal

**Affiliations:** 1 Department of Chemistry, Vanderbilt University, Nashville, Tennessee, United States of America; 2 Department of Pharmacology, Vanderbilt University, Nashville, Tennessee, United States of America; 3 Department of Chemical and Biomolecular Engineering, Vanderbilt University, Nashville, Tennessee, United States of America; University for Continuing Education Krems: Universitat fur Weiterbildung Krems, AUSTRIA

## Abstract

Mood disorders are the leading cause of disability worldwide. Up to 30 percent of individuals with major depressive disorder (MDD) and bipolar disorder (BD) display a seasonal pattern of onset, a phenomenon now recognized in the official diagnostic manuals (DSM-5 and ICD-11). Very little is known about the influence of day length (photoperiod) and sunlight intensity (solar insolation) on seasonal patterns in MDD and BD. Here we report a quantitative approach to examine the relationship between sunlight measures and objectively measured motor activity patterns to understand environmental factors driving seasonality in MDD and BD. Our generalized linear model (GLM) assessment of the Depresjon dataset, which includes short-term (up to two weeks) motor activity recordings of 23 unipolar and bipolar depressed patients and 32 healthy controls recruited to the study at the University of Bergen Norway (60.4° N latitude, 5.3° E longitude), revealed significant association of accelerometer-derived daytime physical activity with participant’s depressed state (p<0.001), photoperiod (p<0.001), and solar insolation (p<0.001). Our study presents a generalizable strategy to decipher the complex interplay between sunlight, physical activity, and depressed state using open-source digital tools. The ability to identify mood disturbances, particularly in seasonally susceptible individuals, using passive digital biomarker data offers great promise in informing next-generation predictive, personalized diagnostics in mental health.

## Introduction

Global prevalence of major depressive disorder (MDD) and bipolar disorder (BD) has recently exceeded 350 million and 60 million, respectively, affecting over 5 percent of the total world population and causing significant disease burden (World Health Organization; Brain and Behavior Research Foundation statistics). A significant fraction (10–30%) of affected individuals exhibit changes in various psychosocial areas of functioning (motivation, sleep, and mood) that are dictated by seasonal patterns of recurrent (hypo)manic and depressive episodes [1–4]. The distinct subtypes of MDD and BDD that are sensitive to seasonal variation, first described as Seasonal Affective Disorder in 1984, can now be diagnosed using the seasonal pattern specifier in the DSM-5 and ICD-11 [3–5]. This marks an important milestone, as being aware of the specific times when one’s energy and mood are affected by the changing seasons can greatly improve self-awareness and help many individuals living with MDD and BD thrive [6].

Humans appear to have a natural ability to track seasonal changes in sunlight duration (photoperiod or daylength) and sunlight intensity (solar insolation—*the amount of solar radiation*, *measured in Watts incident per square meter at a given location*), and there is increasing recognition of the profound, diverse, and complex impact of sunlight on human physiology and behavior [6–8]. Sunlight has been shown to modulate vision, circadian timing, the sleep-wake cycle, neuroendocrine function, alertness, performance, mood, and thermoregulation [7–10]. Our own work in this area suggests that the hypothalamus-pituitary-thyroid (HPT) axis may act as a neuroendocrine mediator between seasonal variations in sunlight and the symptomatology observed in major depressive disorder (MDD) and bipolar disorder (BD) [6, 11, 12]. However, only a few studies have systematically investigated seasonal variations in symptom onset and progression in MDD and BD.

A comprehensive systematic review encompassing 51 studies identified a seasonal pattern in bipolar disorder presentations. Manic episodes exhibited a significant peak during the spring and summer months, while depressive episodes were most prevalent in early winter with a smaller peak potentially occurring in summer as well [13]. A retrospective analysis of admissions to the psychiatric inpatient unit of a major hospital in Turin, Italy, revealed a significant increase in the prevalence of (hypo)manic episodes among patients diagnosed with bipolar disorder (BD) during the months of May to July (N = 730). This period coincides with peak daylight duration, sunlight intensity, and ambient temperature [14]. In a nationwide registry-based study of inpatients admitted to Austrian hospitals between 2001 and 2014 for mania, depression, or mixed episodes (N = 60,607 admissions, 36% male), seasonal variations were observed. Specifically, symptoms of mania were more prevalent during the summer and autumn months, whereas symptoms of depression were more frequent in winter. Mixed episodes of bipolar disorder also exhibited a seasonal trend, with a peak in summer [15]. Studies conducted in Poland and Norway established a clear seasonal pattern of inpatient service utilization for depression with peaks in the months of spring (March to May) and autumn (September to November) [16, 17]. A recent systematic review of a larger number of studies (N = 41) aiming to elucidate seasonal variations in MDD symptoms for both in- and outpatients offered some support for seasonality in clinical depression, although considerable heterogeneity in subjective measures of symptoms was observed [18].

Recently, Bauer *et al*. collected data on BD I patients from geographically disparate sites in both hemispheres across a wide distribution of latitudes [19]. Their rationale was that individuals experiencing greater changes in levels of solar insolation from winter compared to summer months over lifetime would be more susceptible to BD symptom expression. This international team determined that the greater the maximum monthly increase in the amount of solar insolation at the patient’s location at the onset of illness, the younger the age of onset of BD, with a striking 5-year difference in age of onset of BD symptoms between the locations with the largest (i.e. nearer the poles) versus the smallest (i.e. nearer the equator) monthly increases in solar insolation [19]. In a follow-up report, the same team reported a significant inverse relationship between a history of suicide attempts in BD and the ratio of mean winter solar insolation/mean summer solar insolation [20, 21]. A more recent study by the same team reported that the smaller the ratio between the minimum monthly and maximum monthly solar insolation values, the greater the likelihood the first episode polarity was depression in BD I patients [22].

Despite general consensus that symptoms of MDD and BD display a seasonal pattern of fluctuations in many individuals [23], several critical unresolved issues remain—(1) which seasonality parameter fluctuations (daylight duration, daylight intensity, ambient temperature, barometric pressure) are susceptible MDD and BD individuals most sensitive to?; (2) what genetic markers confer individual susceptibility to seasonal variations in symptom expression and program precise seasonal timing of mood state switching in BD and MDD?; (3) which quantifiable phenotypic biomarkers most accurately capture manifestations of seasonal pattern? Significant challenges remain in identifying robust and reproducible biomarkers (*biomarker = an objective genetic*, *biomolecular*, *or morphological indicator of a pathological process used for diagnosis or prognosis*) that are needed to improve BD and MDD patient outcomes [24]. Passive sensor records hold great promise for improved patient-level predictions and a better understanding of BD and MDD pathophysiology [25–27]. The use of wrist-worn actigraphs, a piezoelectric accelerometer that captures periods of activity and rest by measuring gross motor activity (acceleration in 3D), constitutes a scalable, non-invasive, time-sensitive, and cost-effective approach for detecting MDD or BD characterized by notable changes in goal-directed behavior, energy level, movement, and disruption of the sleep-wake cycle [24–27]. Additionally, routine monitoring of activity patterns via smart devices is increasingly feasibly and may enable personalized prediction of clinically relevant symptom changes in mental disorders [26, 28, 29]. According to systematic reviews, the manic state in BD is associated with increased mean motor activity, reduced variability, and augmented complexity of psychomotor behavior, whereas the depressed state in BD and MDD is generally associated with reduced mean motor activity, increased variability and simplicity in activity patterns compared to healthy controls [30–32]. However, the influence of seasonal changes in daylight on motor activity remains ill-defined, particularly in individuals susceptible to seasonal variation in symptoms and residing at higher latitudes with significant seasonal changes in photoperiod and solar insolation.

In our exploratory study, we sought to assess the association of photoperiod and solar insolation with physical activity of depressed subjects versus controls in the open-source Depresjon dataset (available at http://datasets.simula.no/depresjon/). The Depresjon dataset includes short-term (up to two weeks) motor activity recordings (actograms) of 23 unipolar and bipolar depressed patients and 32 healthy controls that were recruited to the study at the University of Bergen, Norway (60.4° N latitude, 5.3° E longitude) [33, 34]. Our secondary focus was to develop a generalizable algorithm based on open-source digital tools for uncovering seasonal patterns in motor activity, a promising biomarker for unlocking clues to mood disturbances and detecting early warning signs of mood disturbances.

## Materials and methods

### Dataset description

The Depresjon dataset consists of motor activity series recorded for 23 unipolar and bipolar depressed patients and 32 healthy controls (23 hospital employees, 5 students and 4 primary care office patients without serious medical or psychiatric symptoms) that were recruited to the study at Haukeland University Hospital at the University of Bergen, Norway (60.4° N latitude) [33, 34]. This dataset was originally collected to investigate motor activity in schizophrenia and MDD, and motor activity was monitored using an actigraph watch worn at the right wrist (Actiwatch, Cambridge Neurotechnology Ltd, England, model AW4). The available metadata for the condition and control groups include timestamp (one-minute intervals), date (date of measurement), activity, sex, age, affective type (BD type I, BD type II, or unipolar), inpatient status, education, employment information, marital status, and Montgomery–Åsberg Depression Rating Scale (MADRS) score [35] at the beginning of the study. The complete description of participants is provided in the original report by Berle and colleagues [33].

### Actogram preprocessing

**Fig 1:**
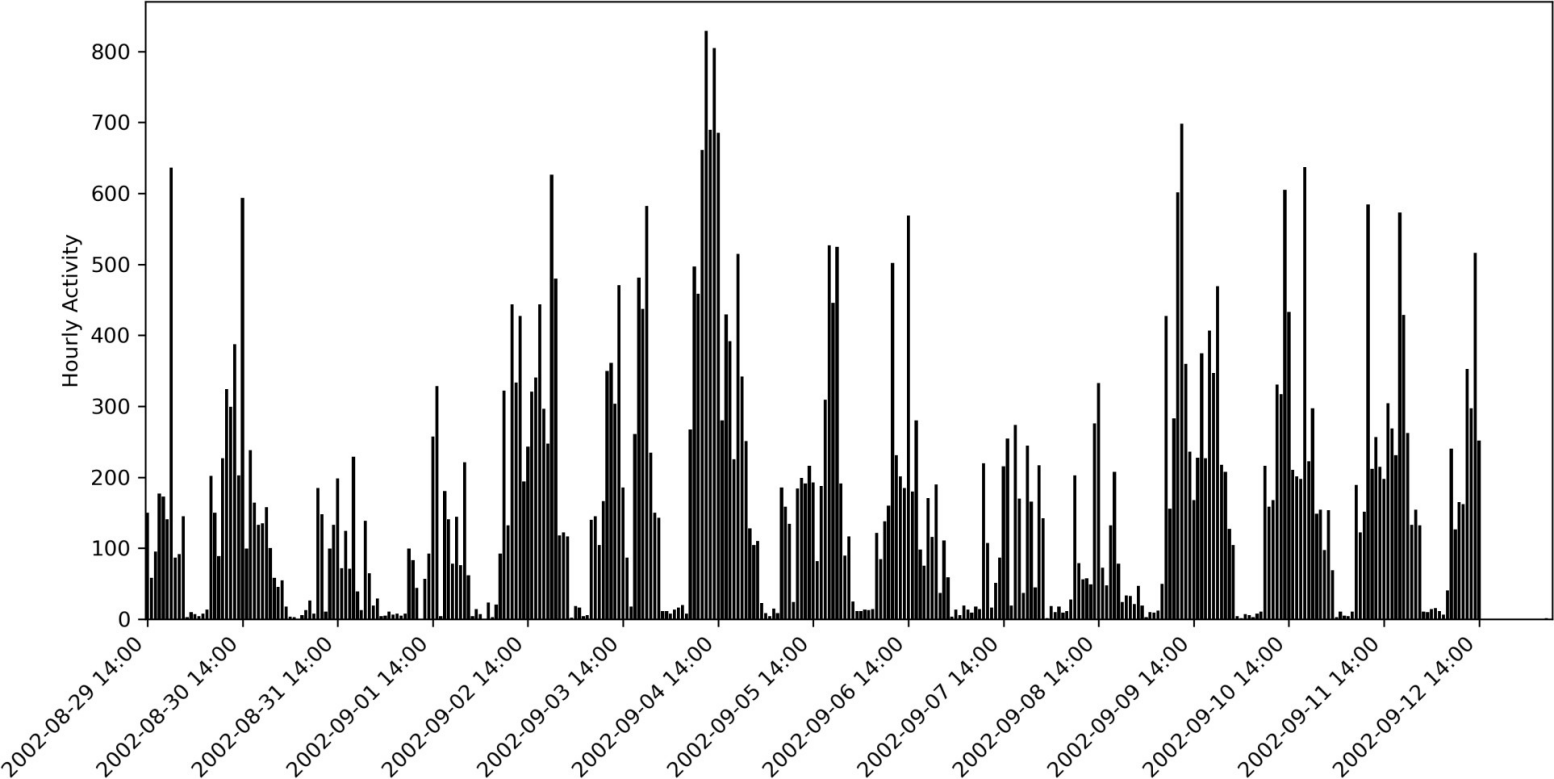
Study participant’s example actogram. A time series of accelerometer-derived hourly acceleration counts is shown as a function of calendar date on the day of accelerometer wear.

The missing motor activity values in the individual actograms were filled with the mean value of a given subject’s activity [36]. Then, the data were resampled with a one-hour frequency and grouped into three subsets for each time series (actogram): full 24 hr data, night data (from 9:00 P.M. to 8:00 A.M.), and day data (from 8:00 A.M. to 9:00 P.M.). An example of an actogram is shown in Fig 1. Next, hourly data for the ‘day’ subset were grouped by the calendar date and averaged to yield a mean hourly activity associated with each calendar date, which was then used to retrieve daily solar insolation and photoperiod data.

### Solar insolation data retrieval

**Fig 2:**
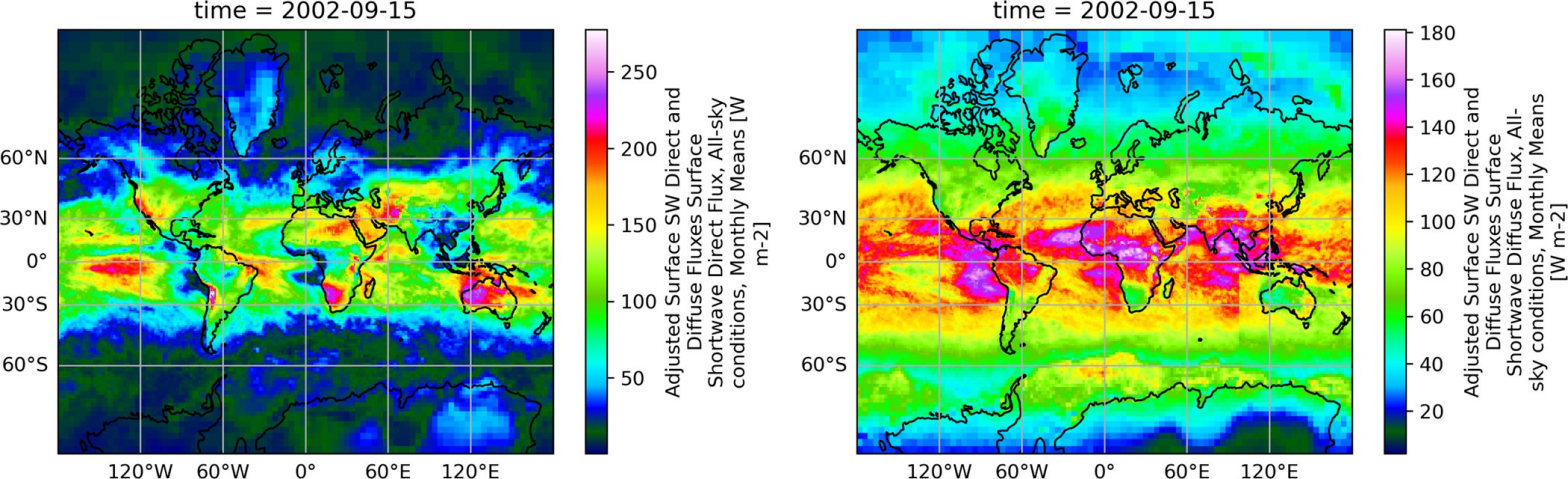
Global surface solar insolation map. Global maps of surface shortwave solar radiation direct flux (left) and surface shortwave diffuse flux (right) under all-sky conditions are shown for September 2002. Made with Natural Earth. Free vector and raster map data @ naturalearthdata.com.

The solar radiation surface flux data for Bergen, Norway were downloaded at daily time resolution as a SYN1deg–Level 3 netCDF4 file using the CERES data ordering tool (https://ceres.larc.nasa.gov/data/). The CERES_SYN1deg-Day_Terra-Aqua-MODIS_Edition4A is NASA’s (The National Aeronautics and Space Administration) Clouds and the Earth’s Radiant Energy System (CERES) and geostationary (GEO)-Enhanced Top-of-Atmosphere (TOA), Within-Atmosphere and Surface Fluxes, Clouds and Aerosols Daily Terra-Aqua Edition4A data product. All-sky surface shortwave diffuse and direct flux data were combined for each calendar date to yield the solar insolation parameter in W/m^2^/day (Fig 2). The maps were generated using an open-source Python library *cartopy* 0.22.0. This library uses Natural Earth public domain maps to generate features, such as land and coastlines. Daily change in solar insolation was computed as the difference in between solar insolation on the current day and solar insolation on the previous day.

### Photoperiod data retrieval

Duration of daylight for each day in a given year was obtained for Bergen, Norway geolocation using the tool developed by the Astronomical Applications Department at the United States Naval Observatory (https://aa.usno.navy.mil/data/Dur_OneYear).

### GLM analysis

General linear models (GLM) were used to examine the relationship between the independent variables (i.e., photoperiod, solar insolation, and change in solar insolation from the previous calendar day) and the dependent variable (average hourly daily physical activity during the day period on a given calendar day). GLM is a flexible statistical model that incorporates normally distributed dependent variables and categorical or continuous independent variables. We employed multivariate GLM for the analysis of accelerometer-derived physical activity measures to include standard confounders (age, sex, and subject depression status at the onset of measurement). GLM was carried out in Python 3.11 using *statsmodels* 0.14.1 module. The following regression formulae were constructed:

formula="activity∼insolation+id+age+sex" (1)

formula="activity∼delta_insolation+id+age+sex" (2)

formula="activity∼hours_of_daylight+id+age+sex" (3)

with *id* being a binary variable indicating the depression diagnosis (0 = healthy control, 1 = depressed subject). The GLM model was selected to be Gaussian with the identity link function. Goodness of fit was monitored based on the calculated *R*^*2*^ value, and the level of statistical significance was set at *p = 0*.*017 (Bonferroni correction for multiple comparisons)*. Additionally, we calculated the variance inflation factor (VIF), a common statistical parameter to evaluate the extent of multicollinearity among independent variables, since a certain degree of correlation between solar insolation integrated over a period of a day and photoperiod is expected. The key difference is that the all-sky solar insolation parameters employed in our study take into account local cloud coverage, aerosol concentration, and other climatic variables. VIF values were determined using the statsmodels 0.14.1 module and yielded the following: VIF_insolation_—4.57, VIF_delta insolation_—1.23, and VIF_photoperiod_—4.31. The general conservative recommendation is that VIF values greater than 5 indicate highly collinear explanatory variables. Therefore, we chose to run 3 separate simple models to better isolate the three explanatory variables for the limited dataset examined. To correct for multiple comparisons testing, we employed Bonferroni correction to adjust the threshold for significance to 0.05/3 = 0.017. To determine interaction between sunlight parameter and depression status, the interaction term (*insolation*:*id* or *hours_of_daylight*:*id*) was added to the formulae used for GLM fitting; backward elimination was used to retain independent input variables with significant association.

### Time-lagged cross-correlation

One-dimensional time series of solar insolation versus calendar date and average hourly activity versus calendar date were combined into a single dataframe for each subject. Augmented Dickey Fuller Test (*adfuller* tool in the *statsmodels*.*tsa*.*stattools* library) was used to check series stationarity. To detrend the time series, differencing was applied with a lag of 1 (first-order derivative) prior to cross-correlation. Time-lagged cross-correlation was determined using the *scipy*.*signal* library (*correlate* and *correlation_lags* tools). Cross-correlation at lag of 0 for depressed versus control subjects was compared using unpaired Student’s t-test, with significance level set at *p = 0*.*05*.

### Ethics statement

This study is a reanalysis of motor activity recordings originating from an observational cohort study presented previously and made available to researchers worldwide at https://datasets.simula.no/depresjon/ [34]. For the original study, the Norwegian Regional Medical Research Ethics Committee West approved the data collection protocol; a written informed consent was obtained from all participants involved in the original study, no compensations for participants in the original study were given, and all processes were in accordance with the Helsinki Declaration of 1975 [33]. Deidentified data available in the public domain were accessed for reanalysis on 9/25/2023. Authors had no access to information that could identify individual participants and did not participate in data collection.

## Results

**Fig 3:**
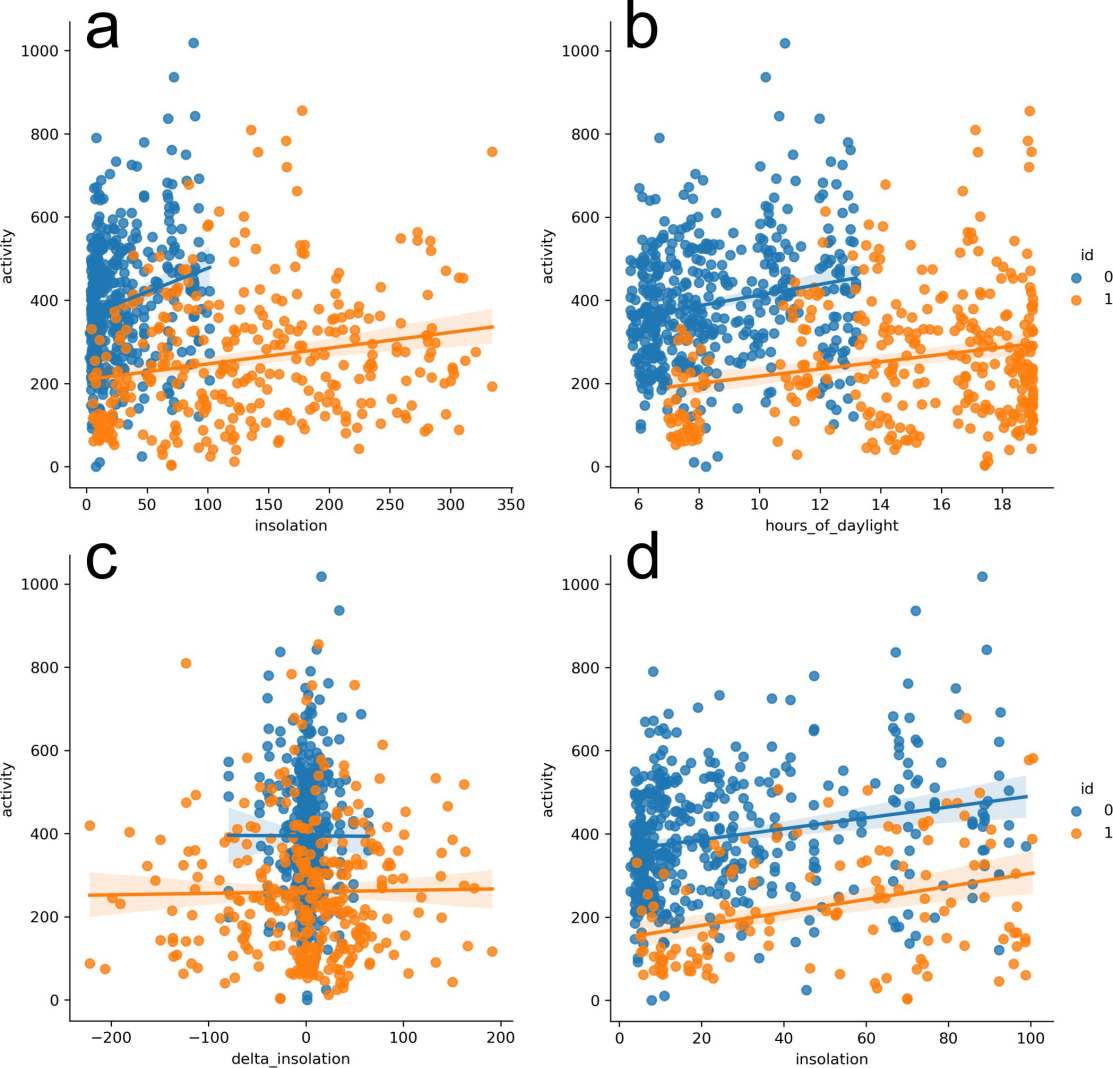
Relationship between sunlight measures and daily activity. Plots of regression model fits are shown for solar insolation versus physical activity (a), photoperiod versus physical activity (b), change in solar insolation from the previous day versus physical activity (c), and solar insolation versus physical activity for insolation *<102 W/m*^*2*^*/day* (d). The plots were rendered using the *lmplot* fuction of the *seaborn* Python library for trend visualization. Blue objects correspond to healthy study participants, and orange objects correspond to depressed subjects. N = 770 total data points derived from 23 depressed subjects and 32 healthy controls.

Dependent variable (average hourly physical activity per calendar day, day portion) data were generated for 55 subjects (23 depressed patients and 32 healthy controls) to yield a total of 770 data points (i.e., a product of 55 subjects × 14 days). These data corresponded to the first 14 days of device wear and excluded days with zero mean hourly activity to correct for non-wear. The dataset with 770 subject-days was converted to a dataframe for subsequent GLM analyses (S1 Data). Summary scatter plots and the corresponding linear regression model line plots of the dependent variable data versus each independent variable are shown in Fig 3. In excellent agreement with previous analyses of the Depresjon dataset [36], Fig 3 revealed a significantly lower daily activity for depressed subjects versus healthy controls. It was also observed that daily activity showed a visually apparent linear correlation with daily solar insolation and photoperiod (hours of daylight) for depressed and healthy subjects. These observations warranted further exploration of the relationship between sunlight parameters and daily activity measure.

### GLM analysis of the association between sunlight and accelerometer-derived activity

Relationships between sunlight parameters (insolation and photoperiod) and daily accelerometer-derived activity were tested using a Generalized Linear Model with the Gaussian error structure (S2 Data). Depression status, age, and sex data were available for all subjects in the Depresjon dataset and were therefore included as covariates in each GLM model. In all models, the depression status (binary covariate) had the greatest statistically significant association with accelerometer-derived activity, with depressed subjects exhibiting significantly lower mean daily activity (Table 1). The Wald z statistic in Table 1 (coefficient estimate divided by the standard error) allowed inter-model comparison and differed less than 3% for the depression status variable across GLM models. Among the sunlight parameters examined, photoperiod had the most significant correlation with daily activity (coefficient estimate: 9.3 ± 1.9, Wald z: 5.0; p<0.001) as well as solar insolation (coefficient estimate: 0.44 ± 0.09, Wald z: 4.9; p<0.001) in contrast to the delta insolation variable (daily change in solar insolation) (coefficient estimate: 0.03 ± 0.12, Wald z: 0.27; p = 0.79) (Table 1). The relative effect (computed as coefficient x σ_photoperiod_/σ_activity_ or σ_insolation_/σ_activity_) was comparable for photoperiod and solar insolation, 0.24 and 0.21 respectively. Participant’s sex (1 = male and 2 = female) had no significant effect on the association for the entire study (depressed and control subjects), whereas participant’s age was positively associated with physical activity (entire study cohort) in the insolation:activity GLM model prior to backward elimination (Table 1). To examine the nature of significant associations in our GLM models, we analyzed the interaction photoperiod/solar insolation with the depression status on physical activity. GLM fitting was run with a corresponding interaction term, and the model was reduced to only include variables with coefficients showing significant association (Table 2). As a result, the interaction of solar insolation with the binary depression status was significantly associated with daytime physical activity (coefficient estimate: -0.76 ± 0.28, Wald z: -2.7; p<0.01) in contrast to the interaction of photoperiod with the depression status (p>0.05). However, this interaction term did not reach statistical significance (p>0.05) for the partial dataset (N = 574) that only included subject-days with solar insolation not exceeding the maximum value (*101*.*6 W/cm*^*2*^*/day*) for control subset (Fig 3, Table 2).

**Table 1:** GLM analysis of the association between sunlight measures and daytime physical activity.

**Solar Insolation (R**^**2**^ **= 0.21)**	**Parameters**	**Coefficient Estimate**	**Standard Error**	**2.5%**	**97.5%**	**Wald *z***	**P**
	**Intercept**	337.01	25.70	286.65	387.38	13.12	<0.001
	**Insolation**	0.44	0.09	0.27	0.62	4.86	<0.001
	**Depression status**	-187.44	14.98	-216.79	-158.08	-12.51	<0.001
	**Age**	1.16	0.46	0.27	2.06	2.55	0.011
	**Sex**	0.25	11.13	-21.57	22.07	0.02	0.98
**Delta Insolation (R**^**2**^ **= 0.19)**	**Parameters**	**Coefficient Estimate**	**Standard Error**	**2.5%**	**97.5%**	**Wald *z***	**P**
	**Intercept**	349.31	25.96	298.42	400.20	13.45	<0.001
	**Delta insolation**	0.03	0.12	-0.20	0.26	0.27	0.79
	**Depression status**	-139.87	11.52	-162.44	-117.31	-12.15	<0.001
	**Age**	1.36	0.46	0.46	2.27	2.95	<0.01
	**Sex**	-5.37	11.24	-27.40	16.67	-0.48	0.63
**Photoperiod (R**^**2**^ **= 0.22)**	**Parameters**	**Coefficient Estimate**	**Standard Error**	**2.5%**	**97.5%**	**Wald *z***	**P**
	**Intercept**	286.54	28.47	230.73	342.35	10.06	<0.001
	**Photoperiod**	9.31	1.86	5.66	12.97	5.00	<0.001
	**Depression status**	-196.36	16.01	-227.73	-164.98	-12.27	<0.001
	**Age**	0.85	0.47	-0.06	1.76	1.83	0.07
	**Sex**	-3.59	11.07	-25.29	18.10	-0.33	0.75

**Table 2:** GLM (interaction) analysis of the association between sunlight measures and daytime physical activity.

**Solar Insolation (R**^**2**^ **= 0.22)**	**Parameters**	**Coefficient Estimate**	**Standard Error**	**2.5%**	**97.5%**	**Wald *z***	**P**
	**Intercept**	362.75	10.12	342.91	382.60	35.83	<0.001
	**Insolation**	1.14	0.26	0.62	1.66	4.33	<0.001
	**Depression status**	-153.92	18.39	-189.97	-117.87	-8.37	<0.001
	**Interaction term**	-0.76	0.28	-1.31	-0.21	-2.71	<0.01
**Photoperiod (R**^**2**^ **= 0.21)**	**Parameters**	**Coefficient Estimate**	**Standard Error**	**2.5%**	**97.5%**	**Wald *z***	**P**
	**Intercept**	307.29	17.13	273.72	340.87	17.94	<0.001
	**Photoperiod**	10.12	1.82	6.56	13.68	5.57	<0.001
	**Depression status**	-198.15	15.78	-229.09	-167.22	-12.55	<0.001
**Solar Insolation < 102 W/m**^**2**^**/day (R**^**2**^ **= 0.28)**	**Parameters**	**Coefficient Estimate**	**Standard Error**	**2.5%**	**97.5%**	**Wald *z***	**P**
	**Intercept**	315.90	19.88	276.94	354.85	15.89	<0.001
	**Insolation**	1.26	0.23	0.82	1.71	5.60	<0.001
	**Depression status**	-202.83	15.04	-232.30	-173.35	-13.49	<0.001
	**Age**	1.17	0.50	0.20	2.15	2.36	0.019

### Time-lagged cross correlation analysis of solar insolation and activity association

**Fig 4:**
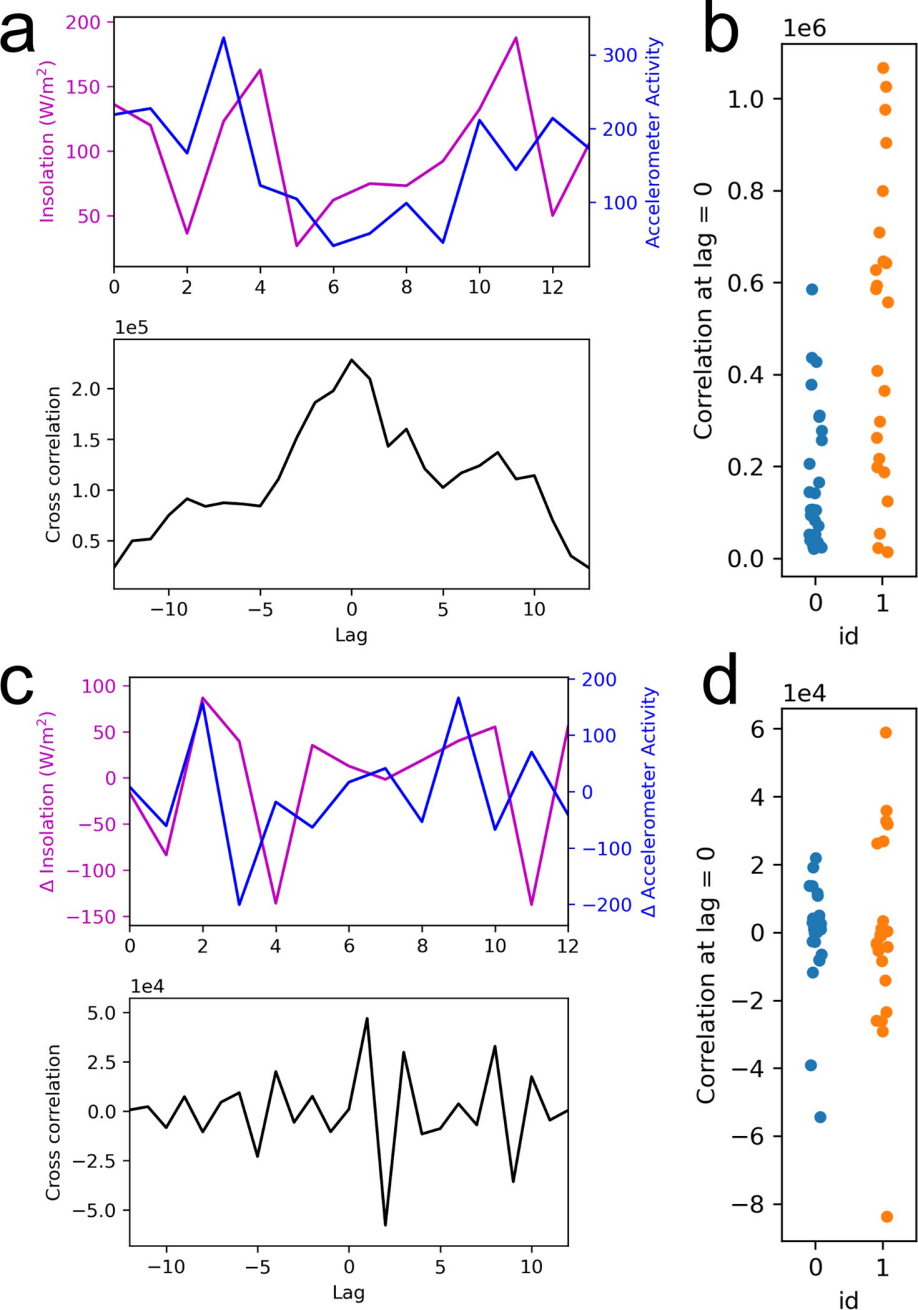
Time-lagged cross correlation analysis of solar insolation versus physical activity time series. Example cross correlation of solar insolation versus daytime physical activity 14-day one-dimensional time series are shown before (a) and after (c) normalization to ensure stationarity via differencing (first derivative). The magnitude of cross correlation is shown for raw data (b) and normalized data (d), with color-coded binary depression status.

In line with the rate-of-change hypothesis, we examined the time-dependent association between solar insolation and physical activity time series, each two weeks long, for every study participant. We employed time-lagged cross correlation analysis; differencing was used to ensure stationarity and normalize time series prior to comparison [37]. Fig 4 shows the distribution of cross correlation as a function of participant’s depression status for raw time series and time series corrected via differencing with a lag of 1 (i.e., first derivative). As expected, cross correlation at lag of 0 for raw time series was significantly greater (p<0.05, Student’s unpaired t-test) for depressed subjects, due to the intrinsic dataset imbalance. In contrast, there was no significant difference in cross correlation at lag of 0 for differenced time series (p>0.05, Student’s unpaired t-test).

## Discussion

In summary, within 55 depressed and healthy individuals wearing a wrist-based activity sensor over the course of two weeks, we quantified the relationship between daily measures of sunlight (photoperiod and solar insolation) and objectively measured physical activity at one-day temporal resolution. Our secondary data analysis study was driven by the pressing need to identify a quantitative biomarker of a psychiatric condition (depression in this case) that can be objectively measured in a low-cost, low-burden, noninvasive manner. Additionally, we sought to explore to what extent motor activity patterns, an increasingly popular digital indicator of mental health, show seasonal susceptibility in depressed subjects compared to healthy controls.

It is important to point out that physical activity and depressed state are characterized by a complex bidirectional relationship. It has been established that reducing sedentary activities is a therapeutic intervention that confers a significant protective effect against the onset of depressive symptoms in MDD and BD [25, 38, 39]; on the other hand, depressive state is associated with lower daytime activity, which may be a proxy marker for depression-specific symptoms, such as sleep disturbances, fatigue, and anhedonia [25, 39, 40]. Our rationale for including two sunlight parameters (day length or photoperiod and sunlight intensity or solar insolation) in our exploratory models is based on a series of recent studies demonstrating i) positive association between day length and physical activity [41], ii) lower depressive state proportion at higher daytime light intensity [42], iii) decreased prevalence of SSRI-treated psychiatric disorders at locations with higher solar insolation [43], iv) contribution of solar insolation to symptom-specific differences in depression [44], and v) previously discussed profound associations of solar insolation with the age of onset, history of suicide attempts, and polarity of the first episode in BD I.

In agreement with prior studies, we found that depressed state was associated with lower accelerometer-derived daytime activity. This was accompanied by positive associations between photoperiod and daytime activity as well as solar insolation and daytime activity, both comparable in magnitude. This result was consistent with previously reported seasonal variations in physical activity at the same location and increased physical activity with greater sunlight exposure in geographically disparate locations [41]. However, if one considers that solar insolation is associated with thermal energy and ambient temperature, this outcome may not hold true for the extremes of daily solar insolation. Rather, an inverted U-shape relationship between solar insolation and physical activity could be expected. Previous studies demonstrated that high thermal stress due to uncomfortable weather conditions (high environmental temperature and humidity) impaired physical activity of athletes and non-athletes alike [45, 46].

Intriguingly, we discovered the interaction between solar insolation and depressed state for the full dataset. The model suggests that the impact of solar insolation on physical activity may differ for depressed and healthy individuals. This finding could arguably indicate that depressed subjects exhibit a compromised physiological link between energy input (solar insolation) and physical activity. On the other hand, it is also possible that increased sedentary behavior results in reduced time spent outdoors and does not allow depressed subjects to capitalize on the benefits of sunlight exposure [47]; one could argue that the efficacy of bright light exposure as an augmentation strategy in depression treatment supports this possibility [48]. A more granular longitudinal analysis did not reveal a significant difference in cross correlation in solar insolation-daytime activity between healthy and depressed participants. Visual assessment of normalized cross correlation (Fig 4) for depressed subjects indicated a trend toward a heterogeneous phenotype; separation was not dependent on the affective type of depression (unipolar or bipolar) or MADRS score at the onset of measurement. However, a larger-scale study conducted at geographically disparate locations with objectively measured sunlight exposure is necessary to confirm our preliminary findings and identify phenotype heterogeneity within each group (e.g., responders versus non-responders; individuals with distinct BD and MDD subtypes susceptible to seasonal patterns). Nonetheless, we describe a generalizable quantitative approach that allows one to decipher the complex interplay between sunlight, physical activity, and depressed state using open-source digital tools.

Overall, the ability to distinguish between depressed and healthy state using passive sensor data offers promise in informing next-generation predictive depression diagnostics. A digital biomarker, like accelerometer-derived motor activity patterns, could form the basis of an early warning system that alerts a clinician to initiate a timely intervention. Incorporating objectively measured sunlight exposure markers (NASA-collected solar insolation data or accelerometer-measured light exposure) could further enhance the predictive power of such tools and lay the foundation for personalized models aimed at individuals susceptible to mood disturbances with seasonal patterns.

### Limitations

There are several important limitations of our study that are discussed below. First, our results indicate detection of association rather than causality; establishing a causal link between sunlight and motor activity patterns or mood disturbances requires statistical power and sample size considerably greater than the sample size of 55 participants in the Depresjon study. Second, the data set provided limited information on confounding variables for all participants (e.g., MADRS score, medication data, received treatment data, body mass index, race, ethnicity). Third, five depressed subjects were in an inpatient facility during actigraph measurement, which likely restricted their mobility and lowered acceleration counts. Finally, the depression status at the study onset was not based on a clinical interview and did not establish either the presence of distinct depressive symptoms or the presence of other co-occurring psychiatric conditions, such as anxiety. Future studies should address these important limitations during the design phase and, if replicated, will support the emerging role of passive digital biomarkers in reducing the socioeconomic burden of mood disorders.

## Supporting information

S1 Data A spreadsheet containing the preprocessed input data for statistical analysis. (XLSX)

S2 Data A spreadsheet containing the output of GLM models. (XLSX)
